# A Mammalian Conserved Element Derived from SINE Displays Enhancer Properties Recapitulating Satb2 Expression in Early-Born Callosal Projection Neurons

**DOI:** 10.1371/journal.pone.0028497

**Published:** 2011-12-08

**Authors:** Kensuke Tashiro, Anne Teissier, Naoki Kobayashi, Akiko Nakanishi, Takeshi Sasaki, Kuo Yan, Victor Tarabykin, Lisa Vigier, Kenta Sumiyama, Mika Hirakawa, Hidenori Nishihara, Alessandra Pierani, Norihiro Okada

**Affiliations:** 1 Graduate School of Bioscience and Biotechnology, Tokyo Institute of Technology, Midori-ku, Yokohama, Kanagawa, Japan; 2 Centre National de la Recherche Scientifique–Unité Mixte de Recherche 7592, Institut Jacques Monod, Université Paris Diderot, Sorbonne Paris Cité, Paris, France; 3 Department of Molecular Biology of Neuronal Signals, Max-Plank-Institute for Experimental Medicine, Göttingen, Germany; 4 National Institute of Genetics, Mishima, Shizuoka, Japan; 5 Bioinformatics Center, Institute for Chemical Research, Kyoto University, Gokasho, Uji, Kyoto, Japan; University of Münster, Germany

## Abstract

Short interspersed repetitive elements (SINEs) are highly repeated sequences that account for a significant proportion of many eukaryotic genomes and are usually considered “junk DNA”. However, we previously discovered that many AmnSINE1 loci are evolutionarily conserved across mammalian genomes, suggesting that they may have acquired significant functions involved in controlling mammalian-specific traits. Notably, we identified the AS021 SINE locus, located 390 kbp upstream of *Satb2*. Using transgenic mice, we showed that this SINE displays specific enhancer activity in the developing cerebral cortex. The transcription factor Satb2 is expressed by cortical neurons extending axons through the corpus callosum and is a determinant of callosal versus subcortical projection. Mouse mutants reveal a crucial function for Sabt2 in corpus callosum formation. In this study, we compared the enhancer activity of the AS021 locus with *Satb2* expression during telencephalic development in the mouse. First, we showed that the AS021 enhancer is specifically activated in early-born Satb2*^+^* neurons. Second, we demonstrated that the activity of the AS021 enhancer recapitulates the expression of *Satb2* at later embryonic and postnatal stages in deep-layer but not superficial-layer neurons, suggesting the possibility that the expression of *Satb2* in these two subpopulations of cortical neurons is under genetically distinct transcriptional control. Third, we showed that the AS021 enhancer is activated in neurons projecting through the corpus callosum, as described for Satb2*^+^* neurons. Notably, AS021 drives specific expression in axons crossing through the ventral (TAG1^−^/NPY^+^) portion of the corpus callosum, confirming that it is active in a subpopulation of callosal neurons. These data suggest that exaptation of the AS021 SINE locus might be involved in enhancement of *Satb2* expression, leading to the establishment of interhemispheric communication via the corpus callosum, a eutherian-specific brain structure.

## Introduction

Retroposons are highly repeated sequences that are dispersed throughout eukaryotic genomes, in which they copy themselves to RNA and integrate back into the genome at a new site by a “copy-and-paste” mechanism [1]–[5]. SINEs (short interspersed repetitive elements) and LINEs (long interspersed repetitive elements) are two major classes of retroposons. Remarkably, the human genome project revealed that ∼42% of the human genome is made up of retroposons [6]. Most transposable elements are non-functional and are commonly regarded at “junk DNA”. At present, however, there are many examples in which retroposons - including SINEs - have acquired function during evolution, a process called exaptation. Exaptation is a kind of adaptation and was originally proposed by Gould and Vrba for cases in which original morphological functions could be altered during evolution to obtain new functions [7]. One such example is feathers, which were originally utilized for insulation but later used for flight. Brosius and Gould also used this term for retroposons that were originally non-functional but later acquired function during evolution [8]. The functions acquired by retroposons are quite diverse. Retroposons are sometimes used as promoters for transcription [9], poly(A) signals, enhancers [10], and silencers [11], [12]. Some retroposons that contain a sequence for a splice donor or acceptor site have been “exonized” to encode amino acids that are part of a protein [13], [14]. One SINE is involved in chromatin modification [15]. In each of these cases, exaptation occurred relatively recently in terms of an evolutionary time scale, and there are very few examples of retroposons that were involved in macroevolutionary processes such as the acquisition of mammalian-specific phenotypes.

In recent years, alignment of many genome sequences has revealed that ∼5% of the human genome is conserved among vertebrates and is subject to purifying selection, and that protein-coding regions comprise only ∼1.5% of the genome [16]. Accordingly, the remaining 3.5% of the human genome corresponds to conserved non-coding elements (CNEs) [17]. CNEs are clade-specific, that is, some are conserved among primates [18] or mammals [19]–[22], others are conserved among amniotes, and others are conserved among all vertebrates [17], [23]–[25]. It should be noted that the number of protein-coding genes is almost the same among all vertebrates (20,000–30,000) and the evolution of such genes is highly conservative, making it difficult to explain morphological macroevolution by changes in protein sequences alone. CNEs are now considered to be a key element responsible for macroevolution or clade-specific phenotypes. In particular, it is presumed that CNEs conserved among mammals are responsible for contributing to mammalian-specific phenotypes such as the placenta, diaphragm, mammary gland, secondary palate, and neocortex [26].

To elucidate the biological significance of CNEs, enhancer analyses based on transgenic assay systems in the mouse have been applied [27]–[29]. Interestingly, these studies have pointed to a strong bias in the activity of CNEs as enhancers in the developing nervous system [27], [28], [30]. In most cases, however, detailed biological and developmental analyses, such as the identification of genes regulated by the enhancer and/or the demonstration of the enhancer's role in developmental processes at the cellular level, are still lacking. Therefore, to fully understand the association between the gain or alteration of transcriptional regulation systems involving CNE enhancer function and its impact on morphological and developmental evolution, it is necessary to combine multi-disciplinary approaches including bioinformatics, extensive profiling of the enhancer function using transgenic mice, and developmental and neurobiological studies.

One of the most interesting discoveries in transposon biology is that some CNEs were derived from ancient transposed elements [31]–[36], indicating that exaptation was also common in the distant past, as first exemplified by amniote-specific SINE1s (AmnSINE1s) [26], [30], [33], living fossil SINEs (LF-SINEs) [31], and mammalian interspersed repeat SINEs (MIR-SINEs) [37]. Surprisingly, it was later shown that at least 16% of eutherian-specific CNEs were derived from transposons [35]. Furthermore, some CNEs derived from SINEs were characterized as *cis*-regulatory elements using mouse transgenic enhancer assays [30], [31], [37]. These findings provide new insights into the contribution of transposable elements to the evolution of various organisms [38] and possible regulatory networks [39], because SINEs are repetitive sequences and can share the same binding sites for potential *trans*-acting factors in different loci of the genome.

Copies of AmnSINE1 are distributed in human, mouse, opossum, and many other mammalian genomes as well as the chicken genome [33]. Remarkably, ∼14% of the AmnSINE1 loci found in the human genome are highly conserved in evolutionarily distant species, most of which are found specifically in mammals as CNEs [33], [40]. This suggests that, after they originated in a common ancestor of amniotes (mammals, birds, and reptiles), more than 100 AmnSINE1 loci were exapted in a common ancestor of mammals (a mammalian-specific exaptation burst) and are now under purifying selection in mammals [26], [30], [33]. Namely, some of these AmnSINE1s obtained functions in the mammalian lineage and have survived for ∼300 million years. Moreover, this mammalian-specific exaptation burst appears to have allowed adaptation to the severe conditions (e.g., superanoxia) that resulted from geological events during the Permian-Triassic mass extinction 250 million years ago [26].

Previously, *lacZ* transgenic mouse systems were used in enhancer assays to test the activities of AmnSINE1 loci [30]. Two of the AmnSINE1 loci, AS071 and AS021, were shown to have enhancer activity consistent with the expression pattern of neighboring genes involved in brain formation [30]. These analyses also showed that the AS021 locus functions as a specific enhancer in the developing cerebral cortex, and its candidate target gene was suggested to be *Satb2*. Satb2 is a transcription factor that binds to the matrix attachment DNA regions [41], [42]. *Satb2* is expressed in the developing neocortex, maxilla, mandible, and skeleton [41], [43], [44] and contributes to the formation of mammalian-specific brain structures such as the corpus callosum and neocortex [42], [45]. Whether the AS021 SINE locus is involved in the regulation of *Satb2* expression during cortical development and in the evolution of mammalian-specific brain structures remains unsolved.

One of the main evolutionary acquisitions of mammals is the six-layered neocortex, which develops from the dorsal telencephalon (pallium), the most anterior part of the brain [46]. In the neocortex, neurons within each layer are generated at similar times and share similar morphology and patterns of connectivity [47]. They can also be subdivided into two major groups: deep-layer and superficial-layer neurons. Superficial-layer neurons are located in layers 2 to 4; those in layer 2 and 3 interconnect different cortical areas by projecting ipsilaterally or contralaterally, whereas those in layer 4 receive the major input connections. Deep-layer neurons are positioned in layers 5 and 6, and the majority sends axons to subcortical targets in the spinal cord, pons, tectum, and thalamus. During neurogenesis, deep-layer neurons (layers 6 and then 5) are generated first (E11.5 to E14.5 in mice), followed by layers 4, 3 and 2 (E14.5 to E17.5). Other vertebrates do not display similar numbers of neurons or layers or this inside-out sequence of positioning [48].

The corpus callosum, a commissural fiber tract, is a structure that is specific to the brain of placental mammals [49]. The corpus callosum and the placenta are probably the most recent acquisitions in mammalian evolution. The vast majority of Satb2-expressing neurons in both superficial and deep layers of the neocortex contribute to the formation of the corpus callosum. Some Satb2-expressing neurons in the deep layers may project axons into other axonal tracts [42]. In addition, *Satb2* directly specifies a callosal neuronal phenotype by suppressing subcortical projection fate through direct repression of *Ctip2* transcription [42], [45].

To characterize the genetic function of the AS021 enhancer, we performed a detailed analysis of the enhancer activity of the AS021 locus *in vivo* and compared it to *Satb2* expression in the developing cerebral cortex. We show that AS021 enhances expression in a specific population of Sabt2-expressing neurons located predominantly in deep layers, paralleling the kinetics of Satb2 expression in these neurons. Our data strongly argue for a role of AS021 in the specific enhancement of *Satb2* expression in deep-layer cortical neurons. This is the first report in which the precise function of a mammalian-specific enhancer derived from a SINE has been analyzed in detail at the cellular level during mouse cortical development.

## Results

### Characterization of the AS021 locus

**Figure 1:**
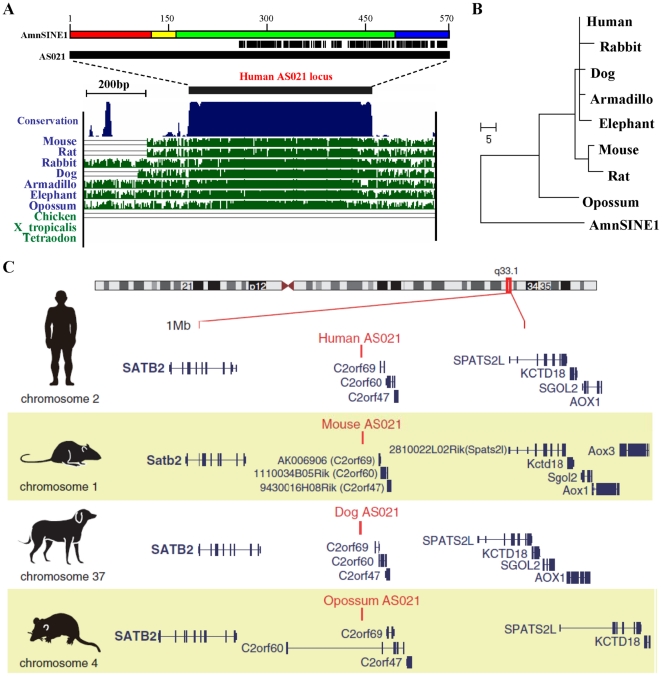
Conservation of the sequence and surrounding gene synteny of the AS021 locus. (A) The human AS021 locus in the UCSC Genome Browser (chr2:200,424,671–200,425,272, hg18). The black bar corresponds to the estimated AmnSINE1-derived region, which is almost identical to the conserved region among mammals. The 3′ half of the AS021 conserved region is homologous to the AmnSINE1 consensus sequence (above, 570 bp), and a sequence alignment of the region is shown in Figure S1. Colors in the AmnSINE1 consensus sequence represent the 5S rRNA–derived promoter (red), tRNA-derived region (yellow), Deu-domain (green), and 3′-tail region (blue). (B) A maximum-parsimony tree using the 3′ half of the AS021 locus from mammals. Note that the internal branch lengths are very short, suggesting the strong selective constraint of the locus. The AmnSINE1 consensus sequence was used as an outgroup. (C) The conserved gene synteny in the region around the AS021 locus (∼1.6 Mb window) among human, mouse, dog, and opossum.

The AS021 locus is a CNE that is conserved among all mammals except monotremes. AS021 locus is not found in the platypus genome, suggesting exaptation of this SINE locus in a common ancestor of Theria (Figure 1). Previously we showed that approximately half of this locus can be aligned with the 3′ half of the AmnSINE1 consensus sequence, the total length of which is 570 base pairs (bp) (Figure 1A, Figure S1). A Maximum-Parsimony tree of the locus shows extremely short internal branches, suggesting a small number of nucleotide differences among species and that the sequence might be under purifying selection during evolution (Figure 1B). Notably, a synteny of genes surrounding the AS021 locus, including *Satb2*, *C2orf69*, *C2orf60*, *C2orf47*, *Spats2l*, *Kctd18*, *Sgol2*, and *Aox1*, is highly conserved among mammals (e.g., human, mouse, dog, and opossum; Figure 1C). To test whether the AS021 locus displays enhancer activity, we previously produced an AS021-HSF51 construct consisting of an 800-bp region containing the mouse AS021 locus, the mouse hsp68 promoter, and the bacterial *lacZ* reporter gene, and we used the construct in transient enhancer assays with transgenic mice [30] (Figure S2). We showed that AS021 drives specific expression of the *lacZ* gene in the dorsal telencephalon starting at E11.5 and progressively expanding to the entire pallium at E13.5 [30]; however, detailed spatiotemporal activity of the enhancer was still unknown.

Here, to further analyze the enhancer activity of the AS021 locus during brain development, we generated stable transgenic mouse lines using the AS021-HSF51 construct (Figure S2). Two lines were established that reproducibly express β-galactosidase (βgal) in the telencephalon as observed in previous experiments using transient transgenic mice [30]. This suggests that the AS021 sequence shows a consistent enhancer function regardless of integration site in transgenic mice. One of these lines was further analyzed by X-Gal staining to determine the time course of βgal expression.

**Figure 2:**
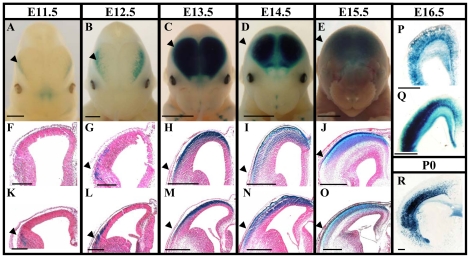
Expression patterns of AS021-lacZ in the telencephalon of transgenic mice during embryogenesis. A–Q. X-Gal staining for β-galactosidase activity on AS021-lacZ embryos at E11.5 (A,F,K), E12.5 (B,G,L), E13.5 (C,H,M), E14.5 (D,I,N), E15.5 (E,J,O), E16.5 (P,Q) and P0 (R) shows a dynamic pattern of expression in the dorsal telencephalon. (A–E) Frontal views of whole-mount stained heads. (F–O) Two coronal sections at rostral (F–J) and caudal (K–O) levels of the corresponding stages. (P–R) Coronal sections at E16.5 (P,Q) and P0 (R). The enhancer activity of AS021 starts at E11.5 in the lateral wall of the caudal telencephalon (prospective piriform cortex; black arrowhead in A,F,K) and progressively expands into the entire pallium between E12.5 and E13.5 (B,C,G,H,L,M). At E15.5–P0, expression is still observed in the entire pallium, within the deeper region of the cortical plate (D,E,I,J,N–Q). Scale bars: 2 mm (A–E), 0.2 mm (F,K), and 0.5 mm (G–J,L–R).

The onset of expression in the telencephalon at E11.5 was observed only in post-mitotic cells in the lateral side of the pallium at caudal levels (Figure 2A,F,K), corresponding to the prospective piriform cortex. At E12.5, βgal expression expanded dorsally into the prospective lateral cortex (Figure 2B,G,L), and by E13.5, it covered the entire pallium including the prospective neocortex (Figure 2C,H,M) following the lateral-to-medial gradient of cortical maturation. Coronal sections at E13.5 revealed expression in the post-mitotic compartment, including the marginal zone (MZ) that will become layer 1 in the mature cortex, and the cortical plate (CP), a densely packed zone of post-mitotic cells [50] beneath the MZ that will become layers 2–6 [51] (Figure 2H,M). Moreover, expression appeared to start beneath the CP in the intermediate zone (IZ) (Figure 2H,M), through which differentiating neurons migrate toward their final location. Similar expression in the cerebral cortex was still observed at later stages of embryonic development (E14.5–E16.5) and at postnatal day 0 (P0) (Figure 2D,E,I,J,N–R), but progressively decreased in the prospective piriform cortex (Figure 2J,O, black arrowhead). Interestingly, βgal expression was also observed in axonal tracts, namely the anterior commissure (ACo) and the corpus callosum (CC), but not in the internal capsule (IC) (Figure 2P–R). No expression was observed outside the cerebral cortex at either E16.5 or P0, for example in the striatum (Str), thalamus, or olfactory bulb (Figure 2P–R and data not shown). Taken together, these results show that AS021 displays specific enhancer activity in the developing cerebral cortex with a precise onset of appearance and spatial distribution in differentiating neurons.

### Comparative analysis of expression between AS021-lacZ and adjacent loci

**Figure 3:**
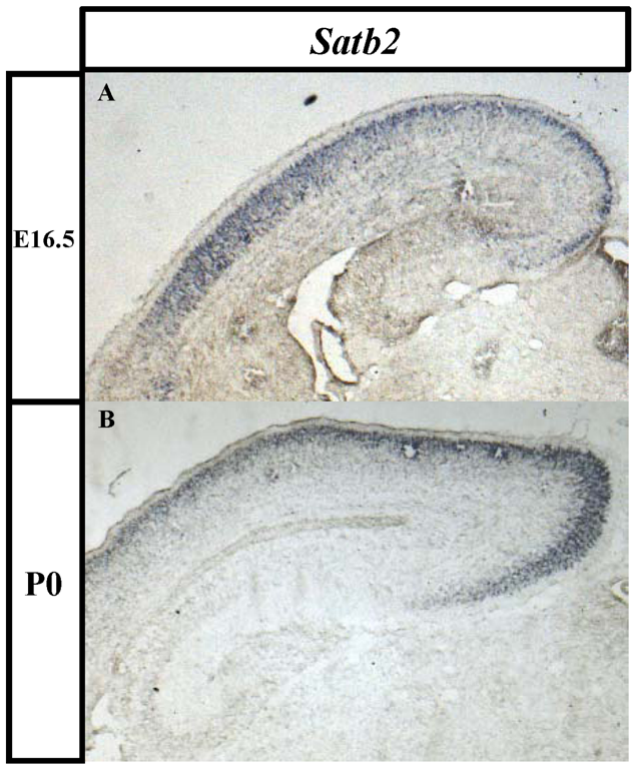
*Satb2* mRNA expression pattern in the developing cerebral cortex. *In situ* hybridization for *Satb2* mRNA in E16.5 (A) and P0 (B) wild-type cerebral cortices. *Satb2* expression is detected in the developing cortical plate but not in the hippocampus.

Genomic comparison throughout mammalian species revealed conservation of a synteny of 13 genes located within 2 Mbp of the AS021 locus, namely *Hsfy2*, *Satb2*, *AK006906/C2orf69*, *11134B05/C2orf60*, *9430016H08/C2orf47*, *DNAPTP6/Spats2l*, *Kctd18*, *Sgol2*, *Aox1*, *Aox4*, *Aox3l1*, *Bzw1*, and *Clk1* (Figure 1). The developmental expression of AS021 was strikingly similar to that of one of these genes, *Satb2* [30]. To test whether any of the other genes also have a similar expression pattern in the developing neocortex, we examined their expression patterns in the telencephalon of E16.5 and P0 animals using *in situ* hybridization. Of these 13 genes, only *Bzw1* (Figure S3 and Table S1) and *Satb2* (Figure 3 and Table S1) were expressed in the telencephalon at E16.5 and P0. A low level of *Bzw1* expression was detected in the developing cortex and in the hippocampus, thalamus, and olfactory bulb (Figure S3 and data not shown). Because the expression pattern for this gene did not correlate with that of AS021-lacZ and because its transcription start site is far (>1 Mbp) from the AS021 locus in the mouse genome, we did not pursue this gene further. Interestingly, the transcription start site of *Satb2* is located 359 and 392 kbp from the AS021 locus in the mouse and human genomes, respectively, and the gene is expressed in the developing cortex with a similar onset and pattern of expression, starting in the prospective piriform cortex at E11.5 [41]. We found that *Satb2* expression progressed dorsally into the CP at later stages and was present throughout the CP during late embryogenesis (Figure 3A,B). Moreover, like AS021-lacZ (see later section), *Satb2* is expressed in neurons projecting through the CC [42], [45].

We conclude that the AS021 enhancer activity correlates both spatially and temporally with the expression of *Satb2* in the developing cerebral cortex, suggesting strongly that the AS021 locus may serve as a cerebral cortex–specific enhancer of *Satb2*.

### Specific expression of AS021-lacZ in a subpopulation of deep-layer Satb2^+^ commissural neurons

**Figure 4:**
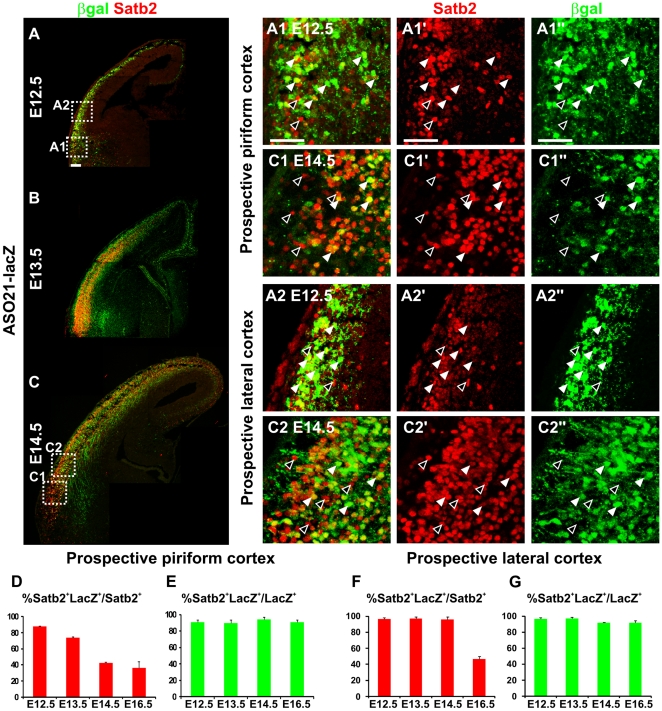
AS021 drives *lacZ* expression in early-born Satb2^+^ neurons. (A–C) Immunostaining for Satb2 (red) and βgal (green) on coronal sections of E12.5 (A-A2″), E13.5 (B), and E14.5 (C-C2″) AS021-lacZ embryos confirms that AS021 drivesβgal expression within Satb2^+^ cells from the earlier stages of *Satb2* expression in the developing cerebral cortex. (A1-A1″,C1-C1″) Enlargement of boxed regions within the prospective piriform cortex in A and C, respectively, showing that most Satb2^+^ neurons also express βgal at E12.5 (white arrowheads) and to a lesser extent at E14.5, as an increased number of Satb2^+^ neurons do not express βgal (black arrowheads). (A2-A2″,C2-C2″) Enlargement of boxed regions within the prospective lateral cortex in A and C, respectively. (D) Graph shows the percentage of Satb2^+^ neurons that co-express βgal (βgal^+^) in the piriform cortex at early stages of cortical development and reveals a decrease in the number of Satb2^+^ neurons that also display AS021 activity during the progression of corticogenesis (87.54±0.65% at E12.5, 74.03±0.64% at E13.5, 42.32±1.07% at E14.5, and 36.43±7.85% at E16.5). (E) Percentage of βgal^+^ neurons that co-express Satb2 in the piriform cortex. The majority of βGal^+^ neurons expresses Satb2 confirming that AS021 drives *lacZ* expression specifically in Satb2^+^ neurons (90.49±2.97% at E12.5, 89.62±3.96% at E13.5, 92.96±3.25% at E14.5 and 90.53±3.03% at E16.5). (F) Percentage of Satb2^+^ neurons that co-express βgal (βgal^+^) in the prospective lateral cortex. On the contrary to the piriform cortex, the fraction of Satb2^+^ neurons also expressing βGal in the lateral pallium is stable from E12.5 to E14.5 (96.97±1.30% at E12.5, 97.30±2.19% at E13.5 and 96.19±3.08% at E14.5) and start decreasing at E16.5 (46.53±6.77%). (G) Percentage of βgal^+^ neurons that co-express Satb2 in the prospective lateral cortex. Similarly to the piriform cortex, AS021 drives specific *lacZ* expression in Satb2^+^ neurons (96.88±1.59% at E12.5, 97.26±1.47% at E13.5, 92.09±0.52% at E14.5 and 91,63±3,08% at E16.5). Graphs show mean ± SEM. Scale bars: 100 µm.

**Figure 5:**
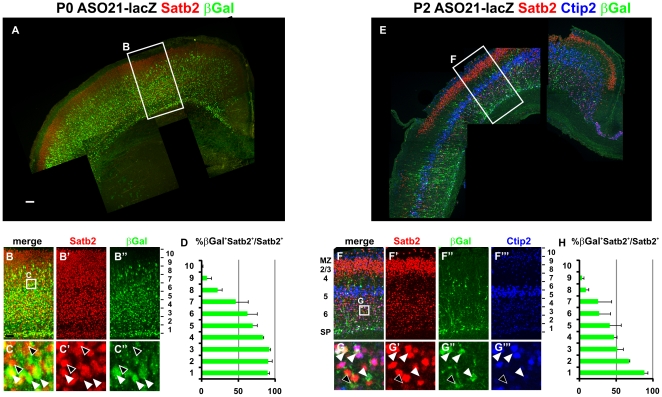
AS021 activity and Satb2 expression decrease within deep layers at postnatal stages. (A–B″) Immunostaining for Satb2 (red) and βgal (green) in coronal sections of P0 AS021-lacZ brains. The cortical plate was divided into 10 equivalent bins, with bin 1 corresponding to the deepest layer and bin 10 corresponding to the most superficial layer. (C-C″) High magnification of boxed region in B showing that only a fraction of Satb2^+^ neurons located in bin 6–7 (corresponding to more superficial layers) also express βgal (white arrowheads). Black arrowheads correspond to Satb2^+^ neurons that do not express βgal. (D) Quantification of the percentage of Satb2^+^ neurons co-expressing βgal (βgal^+^) within the 10 bins. (E–G″′) Immunostaining for Satb2 (red), Ctip2 (blue), and βgal (green) in coronal sections of P2 AS021-lacZ brains reveals a strong decrease of Satb2 staining specifically in deep layers. A similar decrease is observed for βgal, confirming the specificity of the AS021 activity. At both P0 and P2, AS021 activity remains specific to Satb2^+^ neurons (≥90%) in deep layers (D, H). Almost no co-labeling for βgal and Satb2 is observed in bins 8–10, corresponding to prospective layers 2–3 (superficial layers). A fraction of βgal^+^ neurons that expresses Satb2 also express Ctip2 (white arrowheads in G–G″′) in layers 5 and 6. Black arrowheads indicate βgal^+^ neurons that express Satb2 but not Ctip2 in layer 6. For each bin, 10–200 βgal^+^ cells were counted and 200–500 Satb2^+^ cells were counted in at least two animals for each stage. Graphs show mean ± SEM. Scale bars: 200 µm (A), 100 µm (B).

To further analyze the relationship between AS021 enhancer function and *Satb2* expression, we performed co-immunostaining for βgal and Satb2. As shown in Figure 4A–A2″, βgal and Satb2 were co-expressed in the prospective piriform and lateral cortices at E12.5, consistent with the onset of Satb2 protein expression [41]. At E12.5, the vast majority (∼90–97%) of βgal^+^ cells in the CP expressed Satb2 (Figure 4A–A2″,E,G), and most Satb2^+^ cells (∼87–96%) expressed βgal. βgal expression appears to start in the IZ, correlating with the onset of *Satb2* mRNA expression in the IZ [41], [45]. From E13.5, as neurogenesis proceeds in the piriform cortex, the proportion of Satb2^+^ cells co-expressing βgal decreased, but the proportion of βgal cells expressing Satb2 stayed constant (Figure 4B,D,E). The identity of βgal^+^ cells in the prospective neocortex was analyzed using an antibody for Tbr1, a marker of early-born glutamatergic neurons at E13.5. All βgal^+^ cells in both the MZ and CP co-expressed Tbr1 (Figure S4), indicating that they represent early-born glutamatergic cortical neurons. Interestingly, the number of Satb2^+^ neurons not expressing βgal increased at later stages of neocortical development (E16.5), when later-born neurons destined for superficial layers start to populate the CP (Figure 4F,G). By P0, βgal and Satb2 co-expression was mostly detected in deep layers 5–6 of the entire pallium, and to a lesser extent in the more superficial layers 2–4 (Figure 5A–D). Indeed, at this stage, βgal^+^ cells represented 81.15±3.61% of Satb2^+^ neurons in layers 5–6 (bins 1–6 in Figure 5B–D), but only 15.30±4.67% of Satb2^+^ neurons in layers 2–4 and the MZ (bins 7–10). These results indicate that βgal expression driven by the AS021 enhancer correlates with Satb2 expression in early-born but not late-born neocortical neurons.

Interestingly, in the deep layers, Satb2 staining decreased between P0 and P2 along with the reduction of AS021-lacZ activity (Figure 5E–G″ and S5), correlating with the reported reduction of Satb2 expression at P7 [45]. In contrast, strong expression of Satb2, but not βgal, was observed in layers 2–3 at P2. At P21, corresponding to an adult stage, βgal expression was restricted to cells located in the deepest layers of the cerebral cortex (Figure S5). Therefore, the AS021 enhancer recapitulated the expression of Satb2 in deep but not in superficial layers after birth as well as during embryogenesis (Figure 5E–F″). This also confirms that the AS021 enhancer activity is not suppressed in terminally differentiated deep-layer neurons.

**Figure 6:**
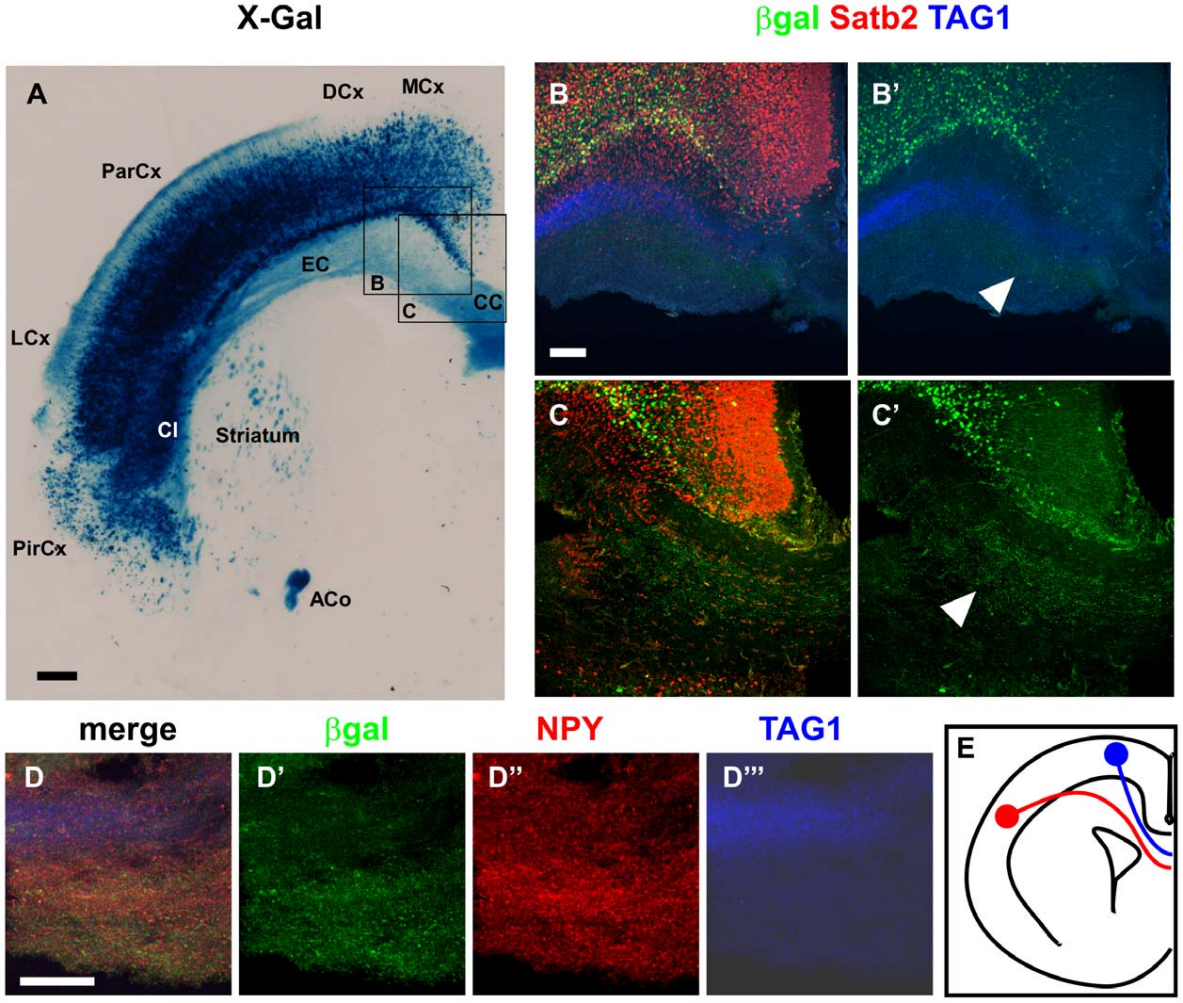
AS021 drives *lacZ* expression in neurons projecting through the corpus callosum. (A) X-Gal staining on 35-µm coronal sections of P0 AS021-lacZ animals shows βgal^+^ projections through the corpus callosum (CC), but also in the external capsule (EC), the striatum, and the anterior commissure (ACo). In addition, projections through the superficial layers can be observed in the parietal (ParCx) and lateral (LCx) cortices but not in the dorsal (DCx) and medial (MCx) cortices. βgal^+^ neurons can be observed in a vast territory of the cortical plate, with the exception of the superficial layers. βgal^+^ cells are also detected in the claustrum (Cl) and in the piriform cortex (PirCx). (B–C′) Immunostaining for Satb2 (red), βgal (green), and TAG1 (blue). TAG1 labels the upper domain of the CC, and βgal^+^ projections are detected within the lower domain of the corpus callosum (TAG1^−^). B and C are single confocal acquisitions from two independent experiments scanned at the approximate levels of the corresponding boxes indicated in A. C corresponds to the same level as A and B to a more rostral section with respect to A. (D–D″′) Immunostaining for βgal (green), TAG1 (blue), and NPY (red). Co-expression of βgal and NPY, which labels the lower domain of the CC, confirms the spatial restriction of βgal^+^ projections to the ventral domain of the CC. (E) Schematic representation shows the relative contribution of axonal projections from the medial and parietal cortices into the dorsal and ventral domains of the CC, respectively [53]. Scale bars: 200 µm (A), 100 µm (B,D).

Moreover, in the prospective layers 5–6 at P2, some βgal^+^/Satb2^+^ neurons also expressed Ctip2, a protein required for formation of corticospinal connections [42], [52] (Figure 5F–G″′, white arrowheads). It has previously been suggested that Satb2^+^/Ctip2^+^ neurons in layer 5 project to either the ACo or cortico-subcortical tracts [42], whereas Satb2^+^/Ctip2^−^ neurons (Figure 5F–G″′, black arrowheads) extend commissural axons toward the CC [42]. Indeed, AS021-lacZ appeared to label axons in the CC and ACo at P0 and P2 (Figure 5A,E and Figure 6A–C′, white arrowheads). We performed co-immunostaining for βgal along with TAG1 and NPY, which stain the dorsal and ventral domains of the CC, respectively [53]. Interestingly, we found that βgal expression co-localized with NPY in the ventral domain of the CC at P0 (Figure 6D-D″), but not with TAG1 in the dorsal CC (Figure 6B–D″′).

Together, these results show that the AS021-lacZ continues to be co-expressed with Satb2 in neurons located in the deep layers at postnatal stages. The vast majority of these neurons project their axons across the ventral CC. All these data support the notion that AS021 might serve as an enhancer for *Satb2* expression in early-generated callosal neurons.

## Discussion

### AS021 SINE enhancer activity recapitulates *Satb2* expression in early-born but not late-born cortical neurons

In this study, we have analyzed the enhancer activity of the AS021 SINE locus in the developing cerebral cortex. We analyzed the 13 genes found within 2 Mbp, and showed that the pattern of lacZ expression driven by AS021 coincides in time and space exclusively with the expression of the Satb2 transcription factor in early-born commissural neurons located in deep layers.

In adults, Satb2 is predominantly expressed in superficial layers of the neocortex, and the vast majority of Satb2^+^ cortical neurons extend axons across the corpus callosum [41], [42], [45]. However, a small subset of Satb2^+^ neurons (∼30%) is also detected in deep layers, where Satb2 can be co-expressed with Ctip2 [42]. Here, we demonstrated that the AS021 SINE element drives expression of the *lacZ* reporter gene in Satb2^+^ neurons in deep layers that project axons into the corpus callosum, but not in Satb2^+^ neurons located in superficial layers. The corpus callosum is divided into ventral and dorsal portions that are innervated by axons from the superficial and deep neocortical layers, respectively (Figure 6E) [53]. Accordingly, we showed that AS021 activated gene expression in Satb2^+^ deep-layer neurons projecting into the ventral CC. From a synteny of 13 genes conserved around the AS021 locus in mammals, none of the other genes were expressed in the developing cerebral cortex. Together, these results show that the AS021 SINE enhancer activity precisely recapitulated *Satb2* expression in a subpopulation of deep-layer commissural neurons. Although these data are still circumstantial evidence, they support the possibility that AS021 serves as a distal enhancer involved in transcriptional control of *Satb2* in these neurons. Moreover, our data may also reflect that expression of *Satb2* is under distinct genetic control in deep versus superficial layers.

### Possible involvement of the AS021 SINE locus in the formation of the corpus callosum through the enhancement of *Satb2* expression

The corpus callosum originated in a eutherian ancestor as a strategy to minimize interhemispheric transmission time for fibers connecting primary and secondary sensory areas [54]. Accordingly, acquisition of the corpus callosum was an important event for the evolution of sensory processing in the eutherian brain. *Satb2* is a key determinant of callosal neuron identity and is required for CC formation. We propose that the exaptation of the AS021 SINE locus might have contributed to the formation of the ventral CC, possibly by enhancing expression of *Satb2* in deep-layer callosal projection neurons.

It should be noted that the AS021 SINE locus is conserved among all therians including the opossum (marsupial), and that the opossum does not have a CC, but instead has interhemispheric connections via the ACo and the hippocampal commissure [54], [55]. It will be interesting to examine the expression of *Satb2* in marsupials and to determine whether it is facilitated by the AS021 enhancer in the neocortex of these species. Nevertheless, the formation of complex structures such as the CC requires the orchestration of multiple cell-autonomous and non-cell-autonomous developmental programs in addition to a *Satb2*-dependent pathway [53], [56], [57]. Moreover, agenesis of the CC has been observed in both mice and humans in mutations other than *Satb2* [56]–[59], strongly suggesting that the enhancement of *Satb2* expression by AS021 in neurons projecting in the ventral CC, even if proven to occur in acallosal marsupials, might not be sufficient to drive CC formation alone in these species. Therefore, in spite of a lack of CC in marsupials, it is expected from the sequence conservation (Fig. S1) that the marsupial AS021 locus has similar enhancer property.

### CNEs involved in gene expression in the developing cerebral cortex are not necessarily related to viability

To elucidate the functions of CNEs including ultraconserved elements, enhancer analyses based on a transgenic assay system in the mouse have been performed in several laboratories [27]–[29]. Furthermore, several knockout (KO) mice that lack ultraconserved elements serving enhancer activities are viable and display no significant abnormalities in macroscopic phenotypes [60]. One interpretation of these results is that the high level of DNA sequence conservation of CNEs does not necessarily correlate directly with biological significance due to the redundancy of such enhancer elements.

The recent discovery in *Drosophila* of “shadow” enhancers with slightly distinct but overlapping activities [61] is interesting from this point of view. Notably, these secondary enhancers located at distant locations contribute to phenotypic robustness in conditions of environmental and genetic variability [62]. Changes in *cis*-acting elements can promote diversity during evolution, but maintenance of essential genetic activities is also crucial. Interestingly, a recent study suggested that a possible shadow (secondary) enhancer of the *Atoh7* gene is responsible for retinal neurogenesis in humans [63]. They proposed that the primary and shadow enhancers may cooperatively act in the expression of *Atoh7* in retinal ganglion cells. Thus, AmnSINEs acting as shadow enhancers might have the potential to evolve new regulatory networks possibly by interacting with existing *cis*-regulatory elements without altering the essential functions of developmental genes.

However, another interpretation of the absence of apparent phenotypic changes in KO mice is possible [60]. *Satb2* mutant mice exhibit small (∼15%) reductions in cortical plate thickness at E18.5 as well as agenesis of the corpus callosum [45]. Superficial layers have been reported to account for 80% of callosal projections, whereas layer 5 contributes only 20% of fibers to the CC [57]. Because the AS021 SINE element is possibly involved in expression of *Satb2* only in deep-layer neurons (∼30% of total Satb2^+^ neurons after P2), phenotypic changes of a KO mouse of this enhancer element are not expected to be detectable by macroscopic analysis, even if the AS021 enhancer is the only element that drives the expression of *Satb2* in deep layers of the neocortex. Moreover, such phenotypic changes are not likely to be related to viability, as even a total lack of the CC in severe pathological conditions in humans and mice does not cause mortality [56], [57], [59]. Our results suggest that therian-specific CNEs that serve enhancer activity in the developing cortex are not likely to be involved in vital functions, but perhaps participate in fine-tuning cortical neuronal subtype identity and connectivity that contributes to the increased complexity of integrative functions and high computational skills in the mammalian brain.

The present study provides the first example in which the enhancer function of a CNE was precisely analyzed from a neurobiological point of view using multi-disciplinary approaches including bioinformatics, mouse genetics, and developmental neurobiology. It might be difficult to evaluate the absence of apparent phenotypic changes in KO mice lacking ultraconserved elements [60] without such a detailed study.

### A concept of the regulation of gene expression by repetitive sequences

It has long been believed that the control of gene expression in vertebrates is of key importance to explaining various biological mechanisms including cell differentiation and morphological evolution; however, most such molecular mechanisms and their evolution remain enigmatic. In 1969, Britten and Davidson proposed an innovative idea regarding mechanisms regulating gene expression in vertebrates [64]. Based on the novel observation that there are a large fraction of repetitive sequences in the genomes of vertebrates such as mammals, which had just started to be recognized at that time, they hypothesized that the generation and propagation of repetitive elements can provide a source of new regulatory DNA. Because of homology in repetitive elements, complex hybrids can form between the DNA strands and/or their transcripts, and these might be involved in higher-order regulation of gene expression. Although several examples of exaptation of SINEs and LINEs (e.g., Alu and L1s) have been reported [65], repetitive sequences such as transposable elements were generally regarded as genomic parasites until recently.

The discovery of CNEs [17], [19], [20], [23]–[25], [31] changed this situation dramatically. Functional studies of CNEs have become an important research direction in comparative genomics, developmental biology, and evolutionary biology [24], [27]–[29]. Using bioinformatics, Bejerano et al. [66] showed that, among the hundreds of thousands of CNEs distributed in mammalian genomes, thousands can be clustered based on sequence similarity. In addition, a number of CNEs contain significant regulatory motifs (such as CTCF [CCCTC-binding factor] insulator sites [67]) and are located close to genes associated with developmental regulatory functions [24]. This implies that many similar kinds of regulatory elements may be distributed throughout mammalian genomes. The origins of such repetitive CNEs might become an important clue to validate and shape Britten and Davidson's model [64], but remained unsolved until recently.

One of the most interesting discoveries in transposon biology is that some CNEs were derived from ancient retroposons [31], [33]. We previously found that AmnSINE1 represents a portion of the repetitive CNEs, most of which are conserved in mammals [33]. This study, together with a study on LF-SINEs [31], suggests for the first time that retroposon insertions can be a source of acquisition of new regulatory networks. A number of mammalian CNEs are known to be derived from transposable elements [32], [34]–[36], and several of these function as enhancers [30], [31], [37]. Thus, a detailed functional analysis of exapted retroposons as shown in this study is more than a concrete demonstration of Britten and Davidson's model. It is also a key milestone toward understanding how alteration or acquisition of gene regulatory mechanisms leads to the evolution of clade-specific morphological traits such as the mammalian neocortex.

### Possible gene regulatory networks derived from AmnSINE1

**Figure 7:**
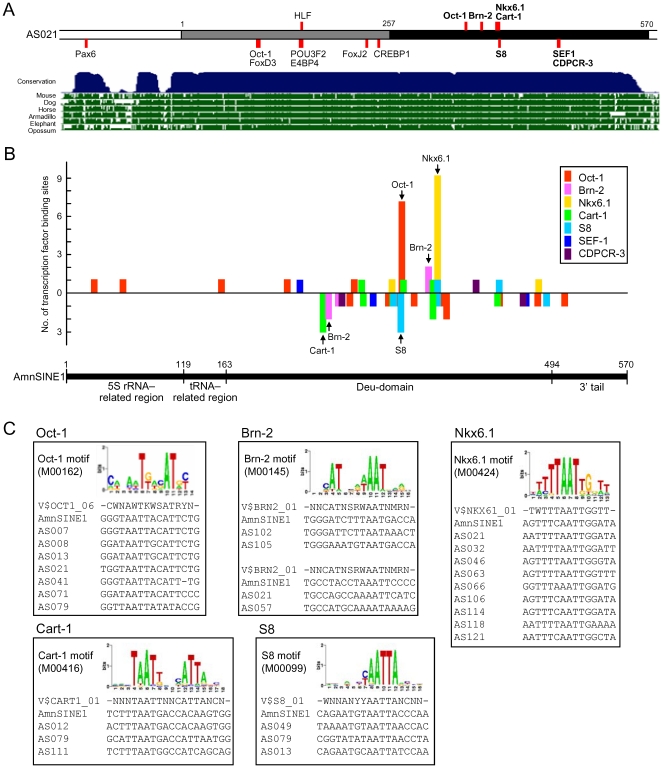
Distribution of transcription factor binding sites in AS021 and other AmnSINE1 loci. (A) Transcription factor binding sites in the conserved region of the AS021 locus. Black and gray bars indicate that the sequence is homologous or not homologous to the AmnSINE1 consensus sequence, respectively. The white bar represents sequences not derived from the SINE. The seven transcription factors in bold were further analyzed in (B). (B) Distribution of the transcription factor binding sites in AmnSINE1-derived CNEs. The binding sites of Oct-1, Brn-2, Cart-1, Nkx6.1, S8, SEF-1, and CDP CR3 were searched in the 124 conserved AmnSINE1 loci collected by Sasaki et al. [30]. Colored bars (Y-axis) represent the number of the transcription factor binding sites identified from the 124 loci in the forward (above) and reverse (below) strands of the corresponding AmnSINE1 consensus sequence (X-axis). Note that most of the binding sites derived from AmnSINE1 are in the Deu-domain. The binding sites, indicated by arrows with names of the transcription factors, were aligned as shown in (C). (C) Sequence alignments of the representative binding sites of five transcription factors (seven OCT-1, four Brn-2, nine Nkx6.1, three Cart-1, and three S8 binding sites), along with the binding motifs obtained from the Transfac database and the AmnSINE1 consensus sequence. The Transfac ID of each motif is shown in parentheses.

The AS021 SINE locus is one of 124 AmnSINE1s that were exapted in a common ancestor of mammals [30]. Because of their repetitive nature, we attempted to address whether possible gene regulatory networks derived from AmnSINE1s exist. The conserved region of the AS021 locus (Figure 7A, gray and black bar) contains 27 transcription factor binding sites that are predicted from conservation in human-mouse-rat alignment and the Transfac matrix database. We focused on the following 15 sites: Pax6, two Oct-1, FoxD3, HLF, POU3F2, E4BP4, FoxJ2, CREBP1, Brn-2, Cart-1, Nkx6.1, S8, SEF-1, and CDPCR-3 (Figure 7A). Interestingly, our enhancer assay for the AS021 sequence with mutations for all 15 binding sites does not show any enhancer activity in the telencephalon of E13.5 embryos (Izawa, Nishihara and Okada, unpublished data) suggesting that at least one of the 15 *trans*-acting factors is responsible for the AS021 distal enhancer activity. This result prompted us to consider the possibility that multiple AmnSINE1 loci, including the AS021 SINE locus, participate in similar regulatory networks by binding to the same *trans*-acting factor(s). To investigate whether other AmnSINE1s in CNEs, as well as the original (consensus) AmnSINE1 sequence [33], contain the same binding sites as the AS021 SINE locus, we focused on the seven binding sites for Oct-1, Brn-2, Cart-1, Nkx6.1, S8, SEF-1, and CDPCR-3 present in the 3′ half of the AS021 SINE locus that has homology with the AmnSINE1 consensus sequence (Figure 7A, black bar). Interestingly, among the 124 conserved AmnSINE1 sequences collected by Sasaki et al. [30], multiple AmnSINE1 loci share the binding sites for Oct-1, Brn-2, Cart-1, Nkx6.1, and S8 in the conserved sequence (Figure 7B). These data suggest that the original (consensus) AmnSINE1 sequence might have retained these binding sites (Figure 7C), having facilitated some of the amplified copies of AmnSINE1s to be exapted in a common ancestor of mammals. These *trans*-acting factors, which are also expressed in the developing mammalian brain, may bind to distant AmnSINE1 loci and may be involved in the same regulatory network of gene expression which was generated at the time of AmnSINE1 exaptation.

Some of the five transcription factors in Figure 7C are expressed in the mammalian central nervous system (CNS). For example, Brn-2 (POU3F2 or Oct-7), a POU domain–containing homeobox transcription factor, has been associated with mammalian brain development. Brn-2 is specifically expressed in neocortical layers 2–5 and can function as an activator as well as a repressor of transcription in different biological systems [68], [69]. Therefore, it is possible that the presence of the Brn-2 binding site in the AS021 locus is involved in the differential expression of *Satb2* between superficial and deep layers of the neocortex. As shown in Figure 7B, four of the 124 exapted AmnSINE1 loci, including AS021, share the Brn-2 binding site. It will be interesting to examine whether these four SINE loci also function as enhancers for a gene(s) specifically expressed in deep layers of the neocortex due to the presence of the Brn-2 binding site. In contrast, Oct-1 (POU2F1), another transcription factor of the POU-homeodomain family, is ubiquitously expressed in both embryonic and adult mouse tissues [70], but its function in cortical development has not been analyzed. Considering that the expression of many developmentally regulated genes is controlled by POU domain–containing transcription factors, it is possible that some of the exapted AmnSINE1 loci are involved in similar regulatory networks through their common binding sites for these factors. Finally, because some of the putative transcription factors binding to the AS021 enhancer are not expressed in the developing cerebral cortex (e.g., Nkx6.1), it is likely that a combination of transcription activators and repressors is responsible for the specific enhancer activity of AS021 in deep-layer Satb2^+^ neurons. Future analysis should elucidate the existence and function of such complex regulatory systems.

### Conclusion

We have shown that the AS021 element may be one of the enhancers for specific expression in Satb2^+^ neurons in the developing pallium. Interestingly, the enhancer activity of AS021 appeared to be restricted to a subpopulation of early-born Satb2^+^ neurons located within deep layers of the cerebral cortex, and was not observed in superficial layers. In addition, we described AS021-driven expression within commissural neurons projecting through the ventral domain of the corpus callosum. These results suggest that AS021 serves as a distal enhancer for *Satb2* transcription in early-born neurons. In that case, we can speculate that there may be differential regulation of *Satb2* expression between the deep and superficial layers. This is the first study in which the enhancer function of a CNE was precisely analyzed from a neurobiological point of view using a multi-disciplinary approach involving bioinformatics, mouse genetics, and developmental neurobiology. This study also provides a milestone for discussion about the involvement of SINEs in the generation of new gene expression networks through shared binding sites for particular transcription factors.

## Materials and Methods

### Ethics Statement

Mouse strains of B6C3F1, C57BL/6 and ICR used in this study were purchased from Sankyo Laboratory Service Corporation (Tokyo, Japan). This study was approved by the Ethics Committee of Tokyo Institute of Technology.

### Mouse transgenic enhancer assay and *in toto* X-Gal staining

The DNA fragment of the AS021 locus was amplified by PCR from MCH mouse genomic DNA [30] and subcloned into the HSF51 vector containing the mouse hsp68 promoter and the bacterial *lacZ* reporter gene (Figure S2; [71]). The construct was linearized with *Sca*I and used for subsequent microinjection experiments as described [72]. Transgene expression of the AS021-HSF51 was determined in F2 embryos by X-Gal staining as described [30]. Genotyping was performed by PCR using genomic DNA samples extracted from yolk sacs of embryos [30]. After X-Gal staining, transgenic embryos at E11.5–15.5 were fixed in Bouin's solution. The fixed embryos were embedded in paraffin for sectioning. After sectioning, tissues were counterstained with eosin and Kernechtrot (Nuclear Fast Red) solution.

### Tissue preparation, immunohistochemistry, X-Gal staining, and *in situ* hybridization

Embryos were fixed by immersion in 4% paraformaldehyde (PFA) in 0.1 M phosphate buffer (PB), pH 7.2 for 2 h at 4°C, and postnatal animals were anesthetized and perfused with 4% PFA in 0.1 M PB for 10 min. Dissected brains were then rinsed in phosphate-buffered saline (PBS) for 2 h, cryoprotected overnight in 30% sucrose/0.1 M PB, and embedded in Tissue-Tek O.C.T.™ Compound (Sakura). Embedded tissues were sectioned on a cryostat with a 12- to 14-µm step for embryonic stages and a 35-µm step for postnatal stages. Fluorescent immunohistochemistry and X-Gal staining were performed as previously described [73]. Primary antibodies were chick anti-βgal (AbCam, 1∶4000), rabbit anti-Tbr1 (Chemicon, 1∶4000), rat anti-Ctip2 (AbCam, 1∶300), and mouse IgM anti-TAG1 (gift from S. Morton and T.M. Jessell, 1∶10). All fluorescent secondary antibodies were purchased from Jackson ImmunoResearch.

For *in situ* hybridization at E16 or P0, NMRI wild-type mice were retrocardially perfused using alkaline phosphate buffer (aPB; 0.1 M Na_2_HPO_4_, pH 9, Merck) containing 10% sucrose (Merck) followed by 5% formalin (diluted from 37% formalin, Merck) in aPB containing 25% sucrose. Mouse brains were isolated, fixed for 3–4 h in 5% formalin in aPB containing 25% sucrose, rinsed in H_2_O, and embedded in Tissue-Tek O.C.T.™ Compound (Sakura). DIG-labeled antisense RNA probes for *in situ* hybridization were synthesized by *in vitro* transcription. Plasmids carrying subcloned coding regions of AS021 surrounding genes were linearized by restriction digestion and incubated for 3 h at 37°C in a 20-µl reaction mixture containing 2 µg linearized template plasmids, 2 µl 10× transcription buffer (Roche), 2 µl DIG-labeled RNA mix (Roche), 0.5 µl RNase inhibitor (New England Biolabs), 2 µl T7 or Sp6 RNA polymerase (Roche), and DEPC-treated H_2_O. cRNA probes were purified by lithium chloride precipitation and monitored by gel electrophoresis.

On the first day, cross-sections (15 µm) were cut using a cryomicrotome and collected on adhesive glass slides (Superfrost Plus, Menzel-Glaeser, Germany). Tissue sections were immediately dried under vacuum for 30 min, fixed in 4% PFA (Merck) in PBS pH 7.4 for 15 min, washed in PBS, and incubated with 20 µg/ml proteinase K (Merck) in 20 mM Tris, pH 7.5/1 mM EDTA, pH 8 for 2.5 min. Proteinase K was inactivated in 0.2% glycine (AppliChem) in PBS. Subsequently, sections were washed in PBS, post-fixed in 4% PFA containing 0.2% glutaraldehyde (Sigma) in PBS for 15 min, washed in PBS, and prehybridized for 2 h at 68°C in hybridization buffer containing 50% deionized formamide (AppliChem), 5× SSC, 1% blocking reagent (Roche), 5 mM EDTA, 0.1% Tween 20 (Sigma), 0.1% CHAPS (Sigma), 0.1 mg/ml heparin (Sigma), and 100 µg/ml yeast RNA (Invitrogen).Sections were then hybridized overnight at 68°C in the same buffer containing denatured probes. On the second day, the slides were washed in SSC, pH 4.5, treated with 20 µg/ml RNase in 0.5 M NaCl/10 mM Tris for 30 min at 37°C, washed in 2× SSC, pH 4.5, washed stringently three times in 50% formamide/2× SSC, pH 4.5 for 30 min each at 63°C, and finally washed three times in KTBT (50 mM Tris pH 7.5, 150 mM NaCl, 10 mM KCl, 1% Triton X-100) for 10 min each. Sections were blocked in 20% sheep serum (Sigma) in KTBT (Ab-block) for 2 h and then incubated with anti-DIG alkaline phosphatase antibody (Roche, 1∶1000 in KTBT overnight at 4°C. On the third day, sections were washed four times in KTBT for 30 min, washed in NTMT (100 mM Tris, pH 9.5, 100 mM NaCl, 50 mM MgCl_2_, 0.1% Tween 20), and developed in NBT/BCIP (Roche) in NTMT. The staining was monitored until signals appeared. The stained sections were subjected to an ascending alcohol series, cleared in 1∶2 benzyl alcohol/benzyl benzoate, and finally mounted using Eukitt (O. Kindler). Unless noted otherwise, incubations were at room temperature and washes were done twice for 5 min each in a slowly shaking (0.25 Hz) cuvette. All solutions used before antibody incubation were prepared using RNase-free (DEPC-treated) H_2_O.

### cDNA sequences and subcloning of cRNA probe templates

The genes surrounding AS021 in different species were identified using the UCSC Genome Bioinformatics Site (http://genome.ucsc.edu/) and were re-examined using the Ensembl Genome Browser (http://www.ensembl.org/index.html). cDNA sequences were collected from the UCSC Genome Bioinformatics Site and re-examined using the NCBI database, and were used to design gene-specific PCR primers (see Table S2) using Primer3 (http://frodo.wi.mit.edu/primer3/) online. Template transcripts were amplified by PCR from an E14 or E17 cDNA pool and then T/A subcloned into T-easy vectors (Promega) according to the product manual. Plasmids were subjected to sequencing to verify the existence and orientation of the appropriate inserts.

### Data collection

Cells labeled by immunofluorescence were counted manually on 12- to 14-µm sections for E11.5 to E16.5 embryonic stages and on 35-µm sections for postnatal stages using ImageJ software.

### Image acquisition

Brightfield images of brain sections were acquired using a Zeiss Axiocam HRc camera coupled to a Zeiss Axiovert 200 microscope. Immunofluorescence images were obtained using an inverted confocal microscope (Leica TCS SP5 AOBS tandem resonant scanner).

### Transcription factor binding sites in conserved AmnSINE1 loci

The 124 conserved AmnSINE1 sequences collected previously [30] were surveyed for predicted transcription factor binding sites using the UCSC Genome database. For seven transcription factors (Oct-1, Brn-2, Cart-1, Nkx6.1, S8, SEF-1, and CDPCR-3) found in the 3′ half of the AS021 SINE locus, we used our own Perl scripts to determine the number of AmnSINE1 loci carrying binding sites at similar locations in the AmnSINE1 consensus sequence [33]. The binding sites found were aligned with the AmnSINE1 consensus sequence using Genetyx software (Genetyx Corporation, Tokyo) to determine their accurate binding sites.

## Supporting Information

Figure S1 **Alignment of AmnSINE1 consensus sequence with mammalian AS021 sequences.** (A) Sequence alignment of the 3′ half of the AmnSINE1 consensus sequence with eight mammalian AS021 sequences. Colors in the AmnSINE1 consensus sequence represent the 5S rRNA–derived promoter (red), tRNA-derived region (yellow), Deu-domain (green), and 3′-tail region (blue). (B) Alignment of the AmnSINE1 consensus and human AS021. The human AS021 locus is homologous to the 3′ half (257–570 bp) of the AmnSINE1 consensus sequence but not to the 5′ half.
(TIF)

Figure S2 **Schematic representation of the transgene construct of the AS021 locus.** (Top) Conservation pattern of the 3.5-kbp region around the mouse AS021 locus. The window of chr1:57,385,565–57,389,078 was obtained from the mm9 assembly at the UCSC Genome Browser (http://genome.ucsc.edu/). The red box indicates the location of the AS021 locus. (Bottom) Schematic representation of the transgene construct of the AS021 locus, showing the fragment subcloned into the HSF51 vector (gray), the mouse hsp68 promoter (dark blue), and the bacterial *lacZ* reporter gene (light blue).
(TIF)

Figure S3 **mRNA expression analysis of genes surrounding the AS021 locus.** (Top, A) Location of the AS021 locus on mouse chromosome 1. (Bottom) Genes surrounding the AS021 locus within a 2-Mbp window. Information about annotated genes was obtained from the UCSC Genome Browser (http://genome.ucsc.edu/). Endogenous mRNA expression patterns of the indicated genes are shown in the telencephalon at E16.5 (Bi–Mi) and P0 (Bii–Mii).
(TIF)

Figure S4 **AS021 drives** ***lacZ*** **expression in early-born glutamatergic neurons.** Immunostaining for Tbr1 (red) and βgal (green) in a coronal section of an E13.5 AS021-lacZ animal. Immunostaining of the individual proteins in the boxed regions of (A) are shown in (B–D″). CP, cortical plate; IZ, intermediate zone; VZ, ventricular zone. Scale bar: 200 µm.
(TIF)

Figure S5 **AS021 activity decreases between P0 and P2.** (A,C) Immunostaining for βgal in coronal sections of P0 (A) and P2 (C) AS021-lacZ animals shows a decrease in the activity of AS021 during early postnatal stages. (B) X-Gal staining at P0 confirms the specificity of the antibody used in (A). (D) X-Gal staining in a coronal section of a P21 AS021 cortex shows staining similar to that observed at P2 in (C), suggesting that the decrease of AS021 activity occurs specifically between P0 and P2. Scale bars: 100 µm.
(TIF)

Table S1 **Expression of genes surrounding the AS021 locus as detected by** ***in situ*** **hybridization in E16 and P0 brains.** P = present; N = not detected.
(TIF)

Table S2 **Primers used for** ***in situ*** **hybridization.**
(TIF)
