# Supplementary figures and images for: A Mammalian Conserved Element Derived from SINE Displays Enhancer Properties Recapitulating Satb2 Expression in Early-Born Callosal Projection Neurons
Source: PLoS One. 2011 Dec 8;6(12):e28497. doi: 10.1371/journal.pone.0028497 (PMC3234267; doi:10.1371/journal.pone.0028497)

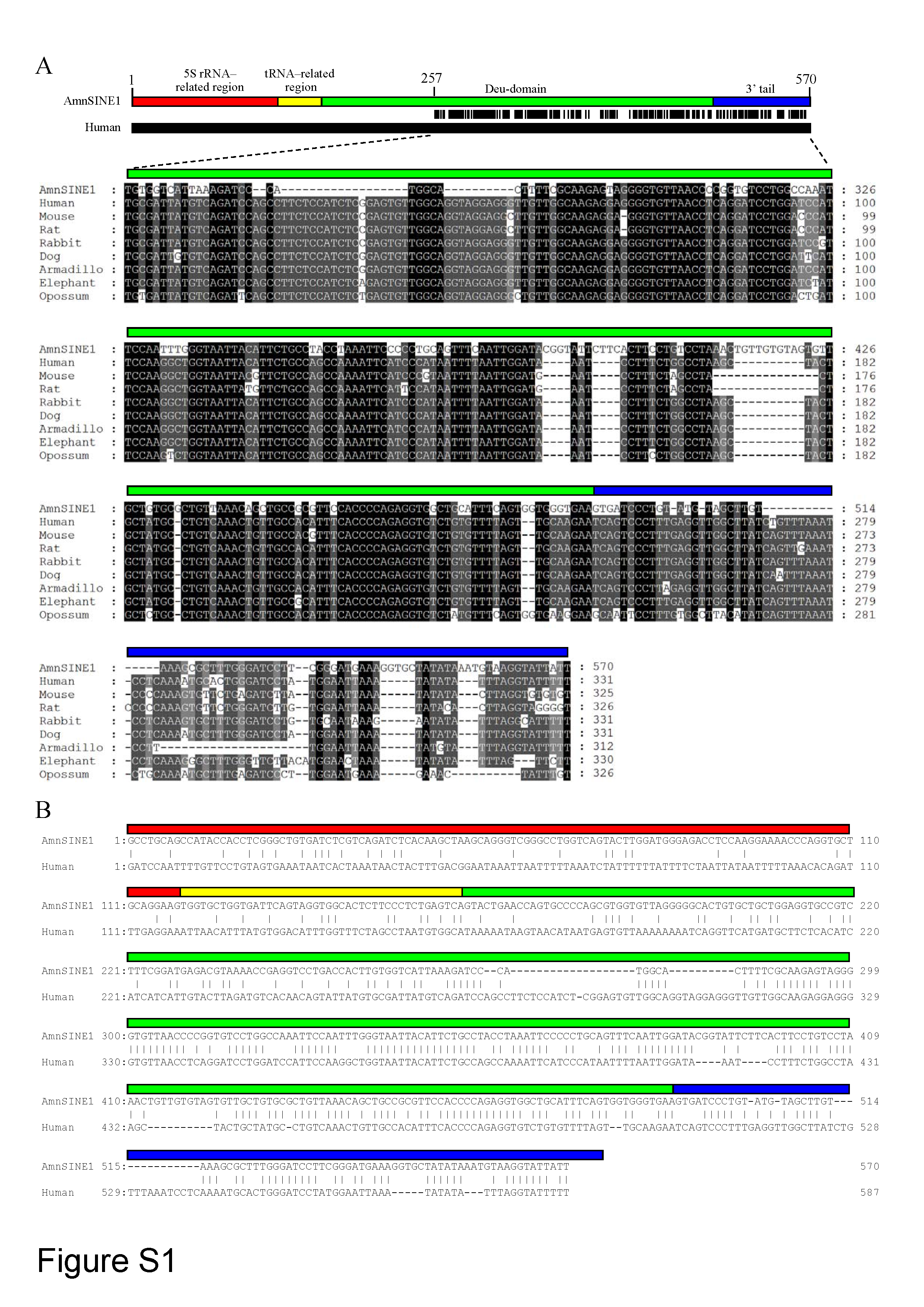

Supplement: Figure S1 — Alignment of AmnSINE1 consensus sequence with mammalian AS021 sequences. (A) Sequence alignment of the 3′ half of the AmnSINE1 consensus sequence with eight mammalian AS021 sequences. Colors in the AmnSINE1 consensus sequence represent the 5S rRNA–derived promoter (red), tRNA-derived region (yellow), Deu-domain (green), and 3′-tail region (blue). (B) Alignment of the AmnSINE1 consensus and human AS021. The human AS021 locus is homologous to the 3′ half (257–570 bp) of the AmnSINE1 consensus sequence but not to the 5′ half. (TIF) [file pone.0028497.s001.tif]

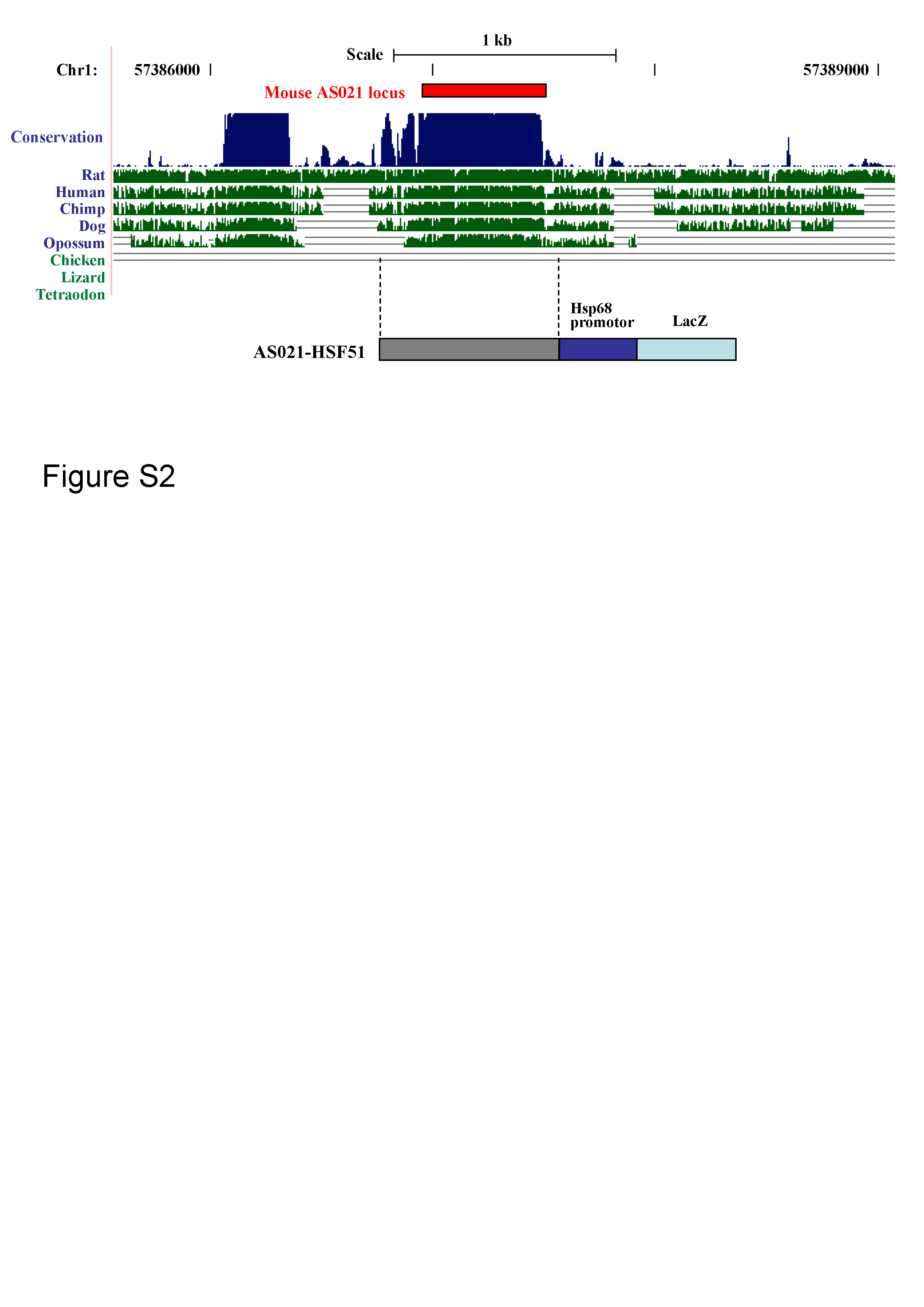

Supplement: Figure S2 — Schematic representation of the transgene construct of the AS021 locus. (Top) Conservation pattern of the 3.5-kbp region around the mouse AS021 locus. The window of chr1:57,385,565–57,389,078 was obtained from the mm9 assembly at the UCSC Genome Browser (http://genome.ucsc.edu/). The red box indicates the location of the AS021 locus. (Bottom) Schematic representation of the transgene construct of the AS021 locus, showing the fragment subcloned into the HSF51 vector (gray), the mouse hsp68 promoter (dark blue), and the bacterial lacZ reporter gene (light blue). (TIF) [file pone.0028497.s002.tif]

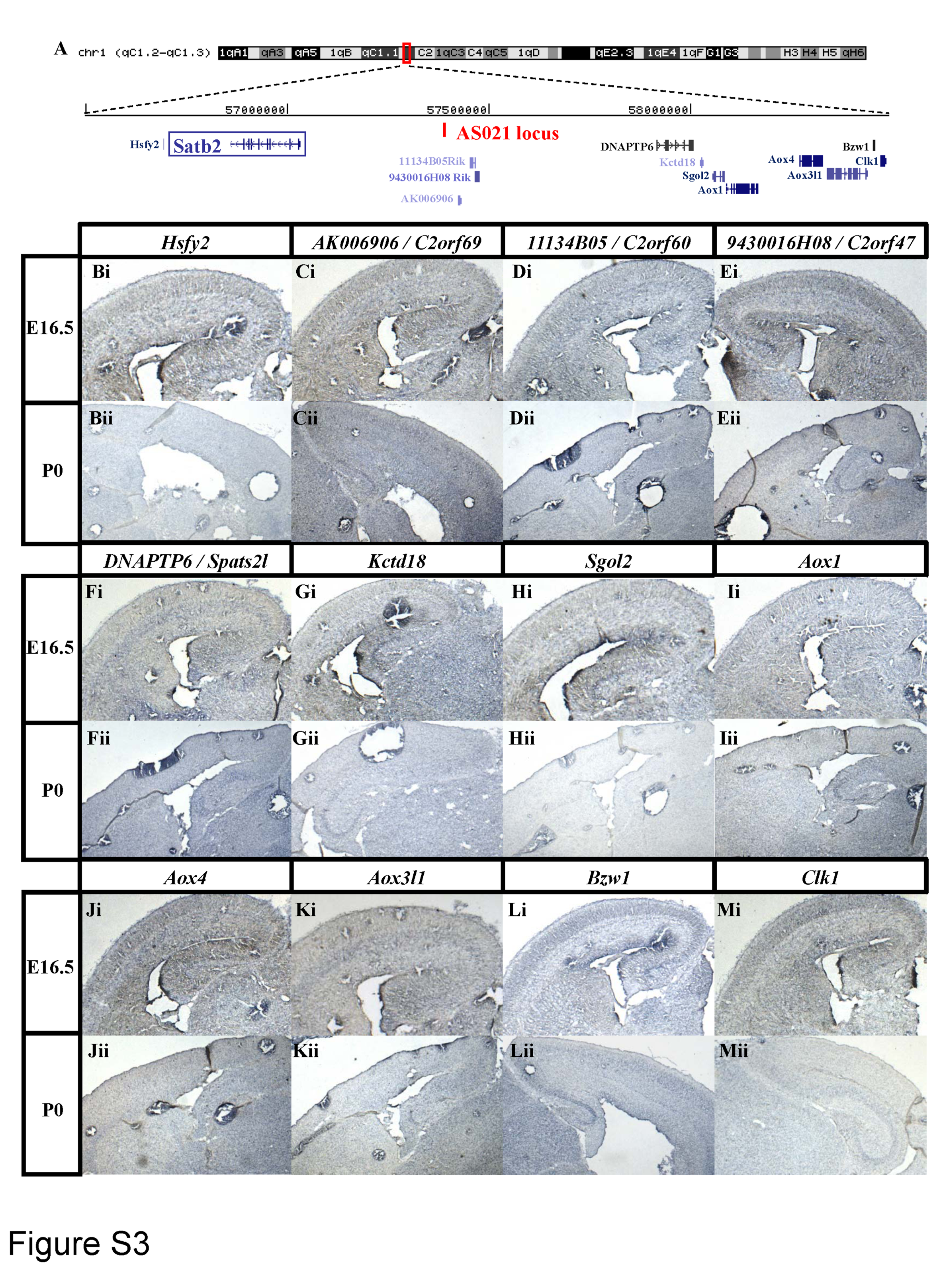

Supplement: Figure S3 — mRNA expression analysis of genes surrounding the AS021 locus. (Top, A) Location of the AS021 locus on mouse chromosome 1. (Bottom) Genes surrounding the AS021 locus within a 2-Mbp window. Information about annotated genes was obtained from the UCSC Genome Browser (http://genome.ucsc.edu/). Endogenous mRNA expression patterns of the indicated genes are shown in the telencephalon at E16.5 (Bi–Mi) and P0 (Bii–Mii). (TIF) [file pone.0028497.s003.tif]

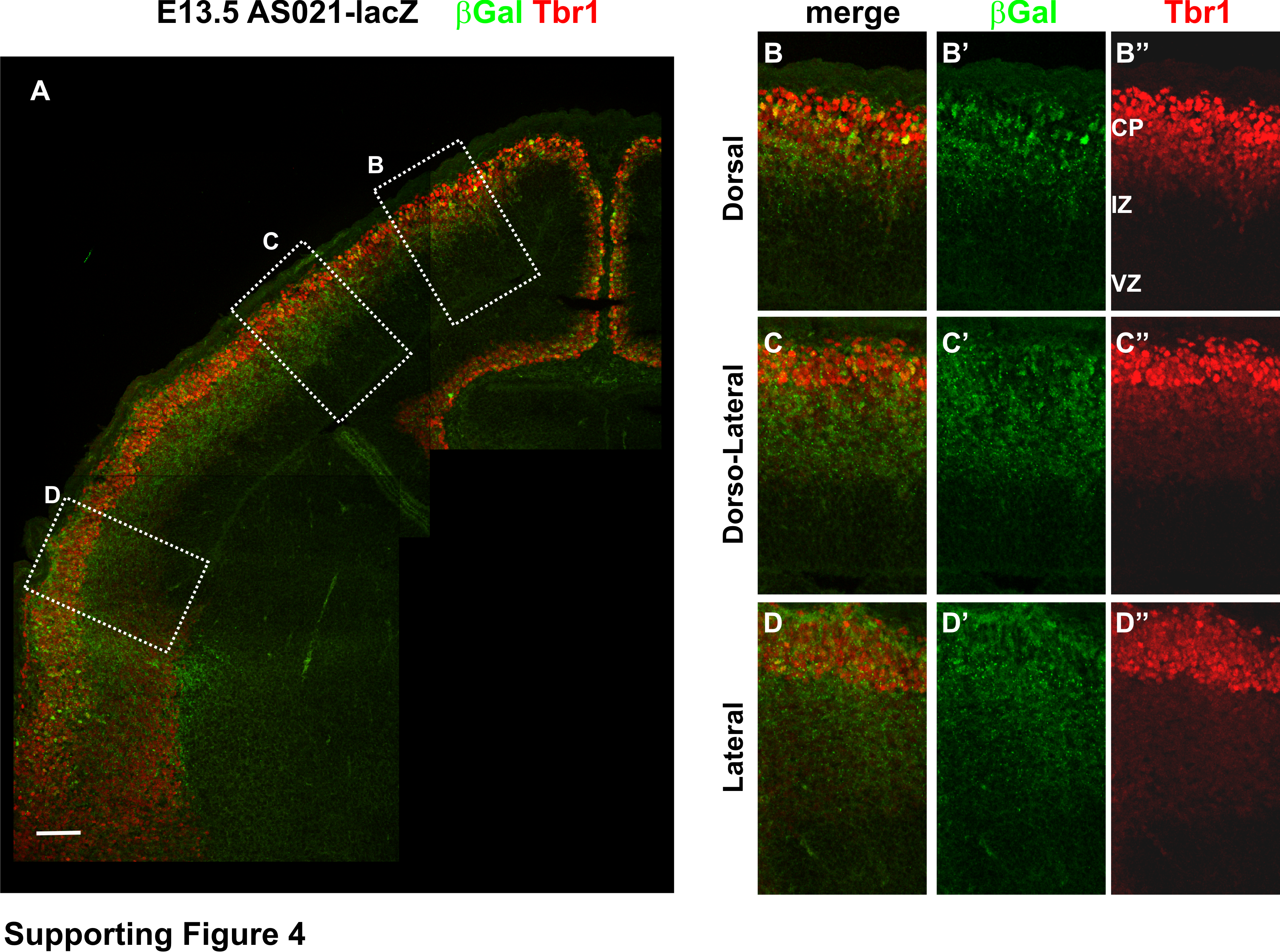

Supplement: Figure S4 — AS021 drives lacZ expression in early-born glutamatergic neurons. Immunostaining for Tbr1 (red) and βgal (green) in a coronal section of an E13.5 AS021-lacZ animal. Immunostaining of the individual proteins in the boxed regions of (A) are shown in (B–D″). CP, cortical plate; IZ, intermediate zone; VZ, ventricular zone. Scale bar: 200 µm. (TIF) [file pone.0028497.s004.tif]

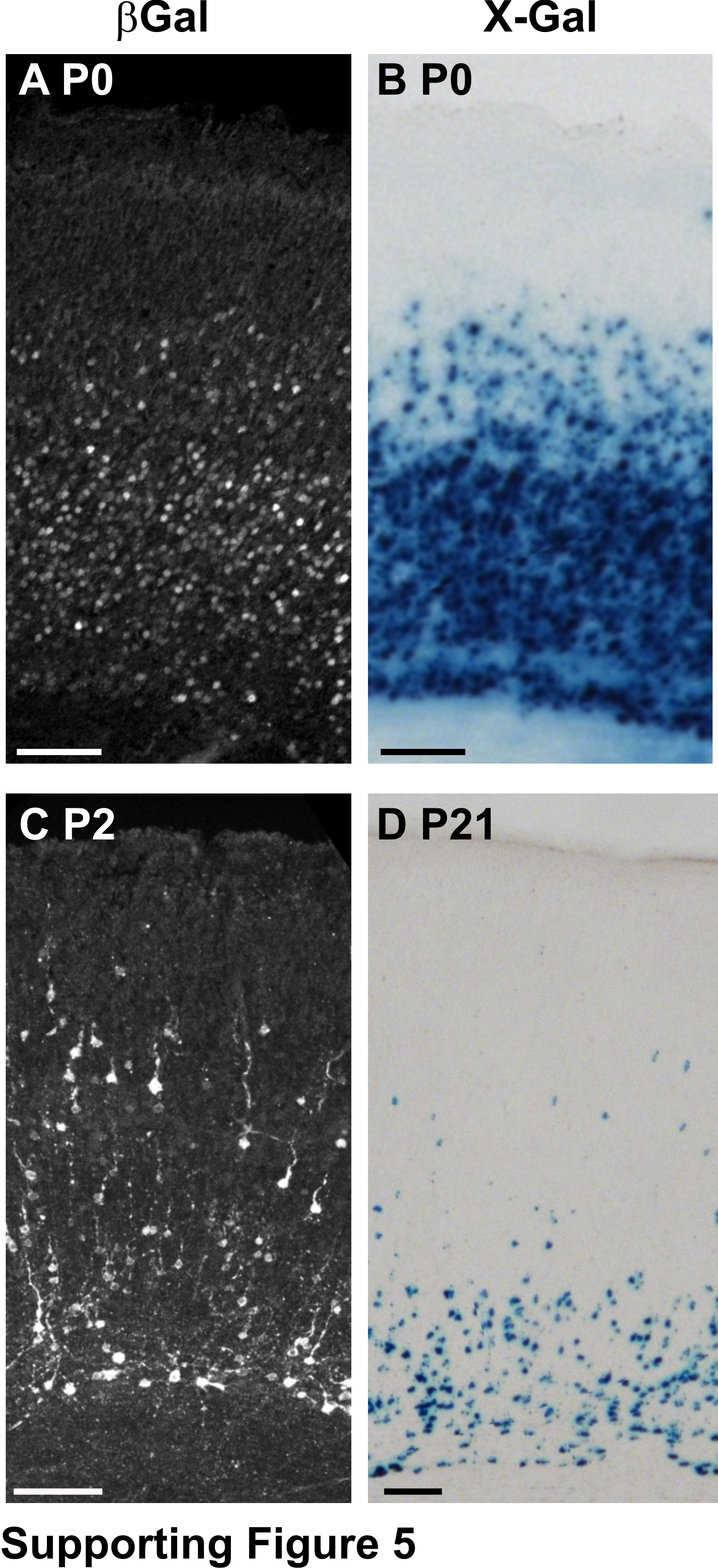

Supplement: Figure S5 — AS021 activity decreases between P0 and P2. (A,C) Immunostaining for βgal in coronal sections of P0 (A) and P2 (C) AS021-lacZ animals shows a decrease in the activity of AS021 during early postnatal stages. (B) X-Gal staining at P0 confirms the specificity of the antibody used in (A). (D) X-Gal staining in a coronal section of a P21 AS021 cortex shows staining similar to that observed at P2 in (C), suggesting that the decrease of AS021 activity occurs specifically between P0 and P2. Scale bars: 100 µm. (TIF) [file pone.0028497.s005.tif]
